# Supplementary material for: Genetic variability and trait associations for physiological and agronomic characteristics in bread wheat genotypes under drought stress and well-watered conditions
Source: PeerJ. 2025 May 16;13:e19341. doi: 10.7717/peerj.19341 (PMC12087580; doi:10.7717/peerj.19341)
Supplement: Supplemental Information 1 [file peerj-13-19341-s001.docx]

Table S1. Mechanical and chemical analysis of the experimental sites in both seasons

| **Characteristics** | **First season** | **Second season** |
| --- | --- | --- |
| **Mechanical analysis** | | |
| Clay (%) | 48.0 | 50.0 |
| Sand (%) | 15.0 | 14.0 |
| Silt (%) | 37.0 | 36.0 |
| Soil texture | Clay | Clay |
| **Chemical analysis** | |  |
| pH | 8.0 | 7.6 |
| EC (dS/m) | 2.5 | 2.2 |
| Organic matter | 1.8 | 1.6 |
| Available P (ppm) | 8.7 | 9.65 |
| Total N (ppm) | 588 | 590 |
| Exchangeable K (ppm) | 443 | 445 |
| **Soil-water constants** | | |
| Field capacity (%) | 37.5 | 36.9 |
| Permanent wilting point (%) | 20.4 | 20.2 |
| Available soil water | 16.5 | 16.3 |

Table S2. Pedigree and origin of evaluated wheat genotypes

| **Code** | **Name** | **Parentage** | **Origin** |
| --- | --- | --- | --- |
| G1 | GEMMEIZA-12 | Bow “s”/Kvz “s”//7C/Seri 82/3/Giza 168/Sakha61 GM 7892-2GM-1GM-2GM-1GM-  0GM | Egypt |
| G2 | SIDS-12 | BUC//7C/ALD/5/MAYA74/ON//1160.147/3/BB/  GLL/4/CHAT"S"/6/MAYA/VUL//CMH74A.630/4*SX | Egypt |
| G3 | L-1117 | CIMMYT/C. 2008/29ESWYT/OCC. 549/Plot141/ Rep1/Block 9/Entry 142 | Mexico |
| G4 | L-1444 | CIMMYT/C. 2008/29ESWYT/OCC. 549/Plot136/Rep1/Block 8/Entry 136 | Mexico |
| G5 | L-123 | SOKOLL/3/PASTOR//HXL7573/2*BAU/4/SRMA/TUI | Mexico |
| G6 | L-125 | PTSS02Y00021S/099B-099Y/099B-099Y/234B-0Y | Mexico |
| G7 | GEMMEIZA-7 | CMH 74A.630 / 5X // SERI 82 /3/ AGENT | Egypt |
| G8 | L-120 | CGSS03B00090T/099Y/099M/099Y/099M/17WGY-0B | Mexico |
| G9 | L-1114 | GEM. 12/ SIDS 12 | Egypt |
| G10 | L-1128 | MS117/ GEM 12 | Egypt |
| G11 | L-1134 | SIDS12/ L-144 | Egypt |
| G12 | L-1142 | GEM12/ L-123 | Egypt |
| G13 | L-1151 | GEM7/ L-125 | Egypt |
| G14 | L-1160 | SIDS12/L121 | Egypt |

Table S3. Stress tolerance indices (averaged over two growth seasons) for 14 bread wheat genotypes under drought and well-watered conditions.

| Genotype | **Mean**  **Productivity** | **Geometric**  **Mean Productivity** | **Harmonic**  **Mean** | **Stress**  **Tolerance Index** | **Yield**  **Index** |
| --- | --- | --- | --- | --- | --- |
| G1 | 5.92 | 5.79 | 5.67 | 0.80 | 0.99 |
| G2 | 4.62 | 4.48 | 4.35 | 0.48 | 0.74 |
| G3 | 6.89 | 6.75 | 6.63 | 1.09 | 1.17 |
| G4 | 6.18 | 5.93 | 5.70 | 0.84 | 0.94 |
| G5 | 5.17 | 5.10 | 5.03 | 0.62 | 0.91 |
| G6 | 6.09 | 6.05 | 6.01 | 0.87 | 1.13 |
| G7 | 5.29 | 5.21 | 5.12 | 0.65 | 0.91 |
| G8 | 6.75 | 6.73 | 6.72 | 1.08 | 1.33 |
| G9 | 5.43 | 5.36 | 5.29 | 0.69 | 0.96 |
| G10 | 5.22 | 5.20 | 5.18 | 0.65 | 1.00 |
| G11 | 5.84 | 5.76 | 5.68 | 0.79 | 1.03 |
| G12 | 5.92 | 5.91 | 5.90 | 0.83 | 1.17 |
| G13 | 4.81 | 4.79 | 4.78 | 0.55 | 0.94 |
| G14 | 4.48 | 4.42 | 4.35 | 0.47 | 0.78 |

Table S4. Direct and indirect effect of studied physiological and agronomic traits on grain yield in wheat under drought conditions

| **trait** | **Chl a** | **Chl b** | **NPR** | **Tr** | **gs** | **RWC** | **MSI** | **MDA** | **Prol** | **CAT** | **APX** | **SOD** | **PH** | **NGPS** | **TKW** |
| --- | --- | --- | --- | --- | --- | --- | --- | --- | --- | --- | --- | --- | --- | --- | --- |
| Chl a | **0.058** | 0.042 | 0.015 | -0.009 | 0.023 | 0.038 | 0.014 | 0.006 | 0.015 | -0.107 | 0.124 | -0.015 | 0.000 | 0.055 | 0.027 |
| Chl b | 0.022 | **0.112** | -0.007 | -0.009 | 0.014 | 0.023 | 0.016 | 0.005 | 0.041 | -0.038 | 0.034 | -0.006 | 0.006 | 0.049 | 0.055 |
| NPR | 0.005 | -0.004 | **0.185** | -0.006 | 0.008 | 0.010 | 0.004 | 0.013 | 0.014 | -0.095 | 0.077 | -0.008 | 0.074 | 0.037 | 0.010 |
| Tr | -0.028 | -0.055 | -0.059 | **0.019** | -0.006 | -0.034 | -0.018 | -0.013 | -0.022 | 0.011 | -0.006 | 0.012 | -0.021 | -0.059 | -0.031 |
| gs | 0.022 | 0.026 | 0.025 | -0.002 | **0.060** | 0.036 | 0.019 | 0.007 | 0.014 | -0.035 | 0.041 | 0.003 | 0.028 | 0.086 | -0.002 |
| RWC | 0.018 | 0.021 | 0.016 | -0.005 | 0.018 | **0.121** | 0.022 | 0.009 | 0.029 | -0.032 | 0.024 | -0.015 | 0.044 | 0.056 | 0.034 |
| MSI | 0.013 | 0.028 | 0.011 | -0.006 | 0.018 | 0.043 | **0.063** | 0.007 | 0.040 | -0.020 | 0.008 | -0.017 | 0.080 | 0.073 | 0.036 |
| MDA | -0.009 | -0.015 | -0.065 | 0.007 | -0.012 | -0.029 | -0.012 | **-0.036** | -0.001 | -0.021 | 0.042 | 0.013 | -0.054 | -0.046 | -0.025 |
| Prol | 0.010 | 0.052 | 0.030 | -0.005 | 0.010 | 0.040 | 0.028 | 0.001 | **0.088** | -0.053 | 0.042 | -0.022 | 0.053 | 0.055 | 0.061 |
| CAT | 0.016 | 0.011 | 0.044 | -0.001 | 0.005 | 0.010 | 0.003 | -0.002 | 0.012 | **-0.395** | 0.438 | -0.013 | 0.038 | 0.031 | 0.037 |
| APX | 0.016 | 0.009 | 0.032 | 0.000 | 0.005 | 0.006 | 0.001 | -0.003 | 0.008 | -0.383 | **0.452** | -0.011 | 0.026 | 0.024 | 0.037 |
| SOD | 0.016 | 0.012 | 0.028 | -0.004 | -0.003 | 0.033 | 0.019 | 0.009 | 0.037 | -0.097 | 0.094 | **-0.053** | 0.078 | 0.081 | 0.054 |
| PH | 0.000 | 0.004 | 0.088 | -0.003 | 0.011 | 0.034 | 0.033 | 0.013 | 0.030 | -0.097 | 0.077 | -0.027 | **0.155** | 0.083 | 0.035 |
| NGPS | 0.019 | 0.033 | 0.041 | -0.007 | 0.031 | 0.041 | 0.028 | 0.010 | 0.030 | -0.075 | 0.065 | -0.026 | 0.078 | **0.164** | 0.040 |
| TKW | 0.011 | 0.045 | 0.013 | -0.004 | -0.001 | 0.030 | 0.017 | 0.007 | 0.039 | -0.108 | 0.122 | -0.021 | 0.040 | 0.048 | **0.136** |

Chlorophyll a (mg/g FW), Chlb: Chlorophyll b (mg/g FW), NPR: Net photosynthetic rate (μmol CO2/m2/s, Tr: Transpiration rate (μmol CO2/m2/s), gs: Stomatal conductance (μmol CO2/m2/s), RWC: Relative water content (%), MSI: Membrane stability index (%), MDA: Malondialdehyde (μmol/g FW), Prol: Proline content (µmol/g DW), SOD: Superoxide dismutase (unit mg/ protein), CAT: Catalase (unit mg/ protein), APX: Ascorbate peroxidase (unit mg/ protein), PH: Plant height (cm), NGS: Number of grains /spike, TGW: 1000-grain weight (g), and GY: Grain yield (tons/ha).

**Table S5. G**enetic variability parameters for physiological and agronomic characters in evaluated genotypes under normal (NOR) and water deficit (DRO) conditions.

| **Parameter** | **Irrigation** | **Chl *a*** | **Chl *b*** | **NPR** | **Tr** | **gs** | **RWC** | **MSI** | **MDA** |
| --- | --- | --- | --- | --- | --- | --- | --- | --- | --- |
| σ²g | Nor | 0.01 | 0.15 | 9.62 | 0.08 | 0.02 | 19.11 | 12.22 | 46.04 |
|  | Dro | 0.02 | 0.04 | 2.49 | 0.32 | 0.01 | 7.58 | 38.45 | 7.43 |
| σ²p | Nor | 0.02 | 0.18 | 10.28 | 0.21 | 0.02 | 31.23 | 20.50 | 51.42 |
|  | Dro | 0.03 | 0.05 | 2.82 | 0.41 | 0.01 | 20.16 | 49.66 | 11.13 |
| GCV | Nor | 1.54 | 19.40 | 15.63 | 7.03 | 19.27 | 5.55 | 6.63 | 18.47 |
|  | Dro | 5.19 | 12.70 | 10.44 | 18.13 | 20.52 | 4.63 | 14.64 | 5.02 |
| PCV | Nor | 4.06 | 20.72 | 16.15 | 11.02 | 19.63 | 7.09 | 8.59 | 19.52 |
|  | Dro | 6.19 | 13.89 | 11.12 | 20.46 | 22.37 | 7.55 | 16.64 | 6.14 |
| H²b | Nor | 14.46 | 87.73 | 93.61 | 40.63 | 96.32 | 61.18 | 59.63 | 89.52 |
|  | Dro | 70.25 | 83.68 | 88.08 | 78.55 | 84.14 | 37.60 | 77.42 | 66.76 |
| GA | Nor | 0.04 | 0.76 | 6.18 | 0.38 | 0.26 | 7.04 | 5.56 | 13.22 |
|  | Dro | 0.23 | 0.37 | 3.05 | 1.03 | 0.19 | 3.48 | 11.24 | 4.59 |
| **Parameter** | **Irrigation** | **Proc** | **CAT** | **APX** | **SOD** | **PH** | **NGPS** | **TKW** | **GY** |
| σ²g | Nor | 0.01 | 0.95 | 2.11 | 4.29 | 96.25 | 51.91 | 10.71 | 0.70 |
|  | Dro | 0.03 | 4.28 | 11.66 | 17.23 | 82.91 | 31.04 | 7.59 | 0.44 |
| σ²p | Nor | 0.02 | 1.47 | 2.39 | 10.32 | 116.58 | 62.56 | 37.65 | 1.58 |
|  | Dro | 0.03 | 5.17 | 14.46 | 22.84 | 93.96 | 43.14 | 9.91 | 1.16 |
| GCV | Nor | 6.17 | 10.16 | 20.06 | 5.59 | 10.47 | 11.26 | 7.01 | 12.93 |
|  | Dro | 9.66 | 13.63 | 25.75 | 6.58 | 12.17 | 10.49 | 7.78 | 13.99 |
| PCV | Nor | 6.83 | 12.60 | 21.31 | 8.68 | 11.52 | 12.36 | 13.15 | 19.44 |
|  | Dro | 10.02 | 14.98 | 28.67 | 7.58 | 12.96 | 12.36 | 8.89 | 22.61 |
| H²b | Nor | 81.54 | 65.05 | 88.58 | 41.53 | 82.56 | 82.98 | 28.46 | 44.25 |
|  | Dro | 92.98 | 82.81 | 80.61 | 75.41 | 88.24 | 71.95 | 76.60 | 38.30 |
| GA | Nor | 0.07 | 1.62 | 2.82 | 2.75 | 18.36 | 13.52 | 3.60 | 1.15 |
|  | Dro | 0.34 | 3.88 | 6.32 | 7.43 | 17.62 | 9.74 | 4.97 | 0.85 |

σ²p: Phenotypic variance, GCV: genotypic coefficient of variation, σ²g: Genotypic variance, PCV: phenotypic coefficient of variation, H²b: Broad-sense heritability, GA: Genetic Advance.


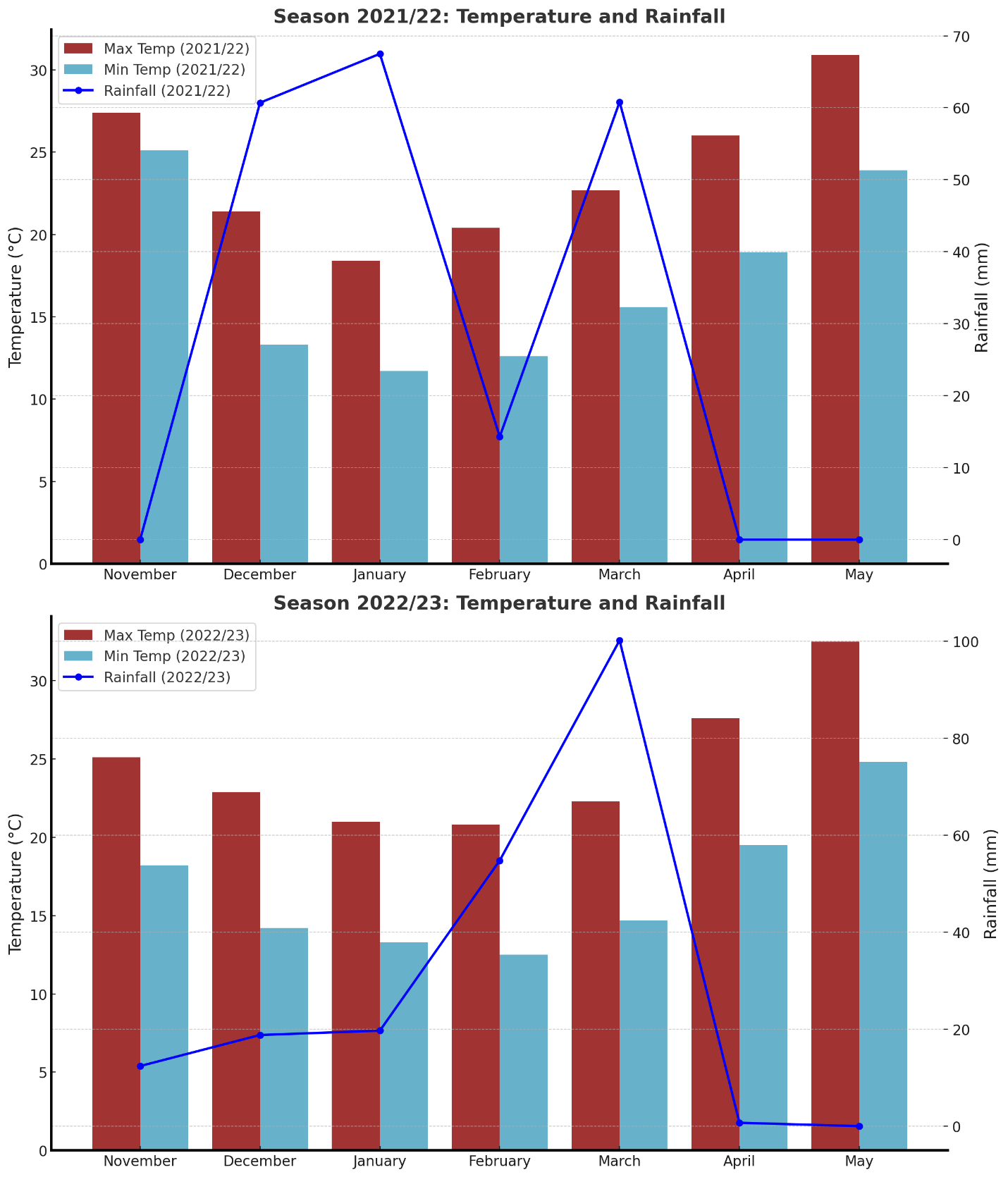


**Figure S1.** Meteorological data of the experimental site illustrating maximum and minimum temperatures, as well as precipitation levels, recorded across the two growing seasons.


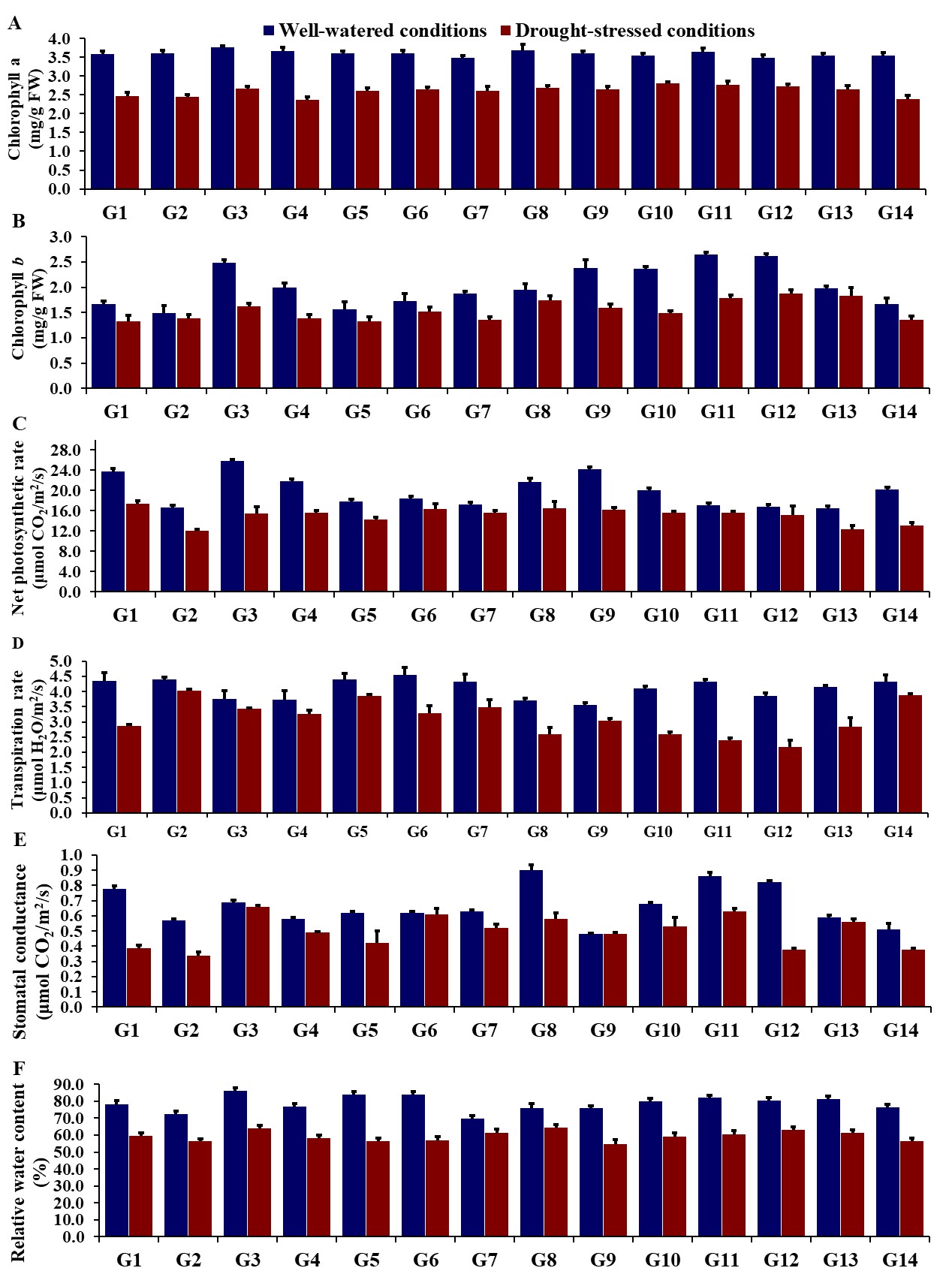
**Figure S2**. Comparative performance of evaluated wheat genotypes: (A): Chlorophyll *a*, (B): Chlorophyll *b*, (C): Net photosynthetic rate, (D): Transpiration rate, (E: Stomatal conductance, and (F): Relative water content. The standard error (SE) is shown by the bars above the columns.

**
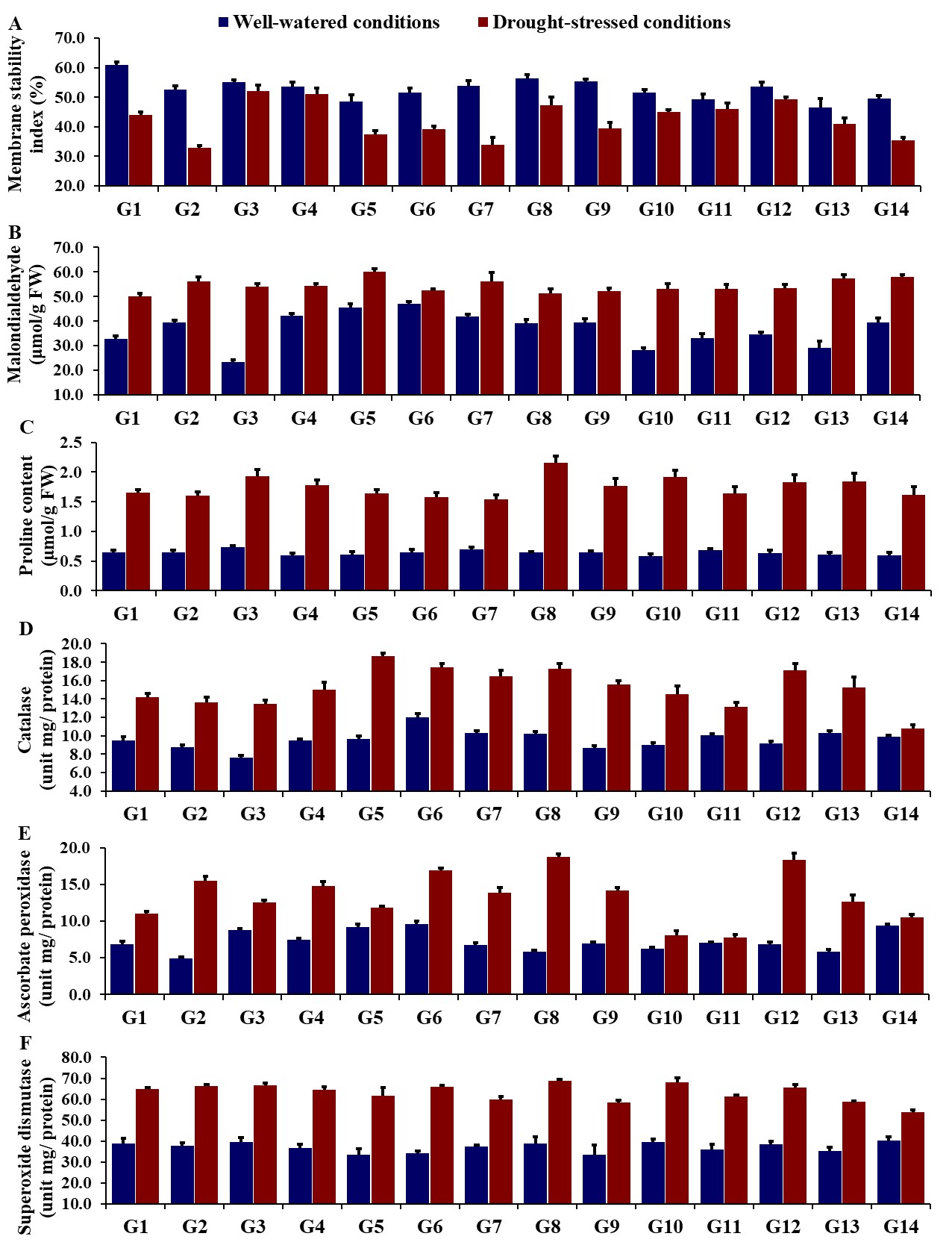
Figure S3**. Comparative performance of evaluated wheat genotypes: (A): Membrane stability index, (B): Malondialdehyde, (C): Proline content, (D) Catalase, (E): Ascorbate peroxidase and (F): Superoxide dismutase. The standard error (SE) is shown by the bars above the columns.


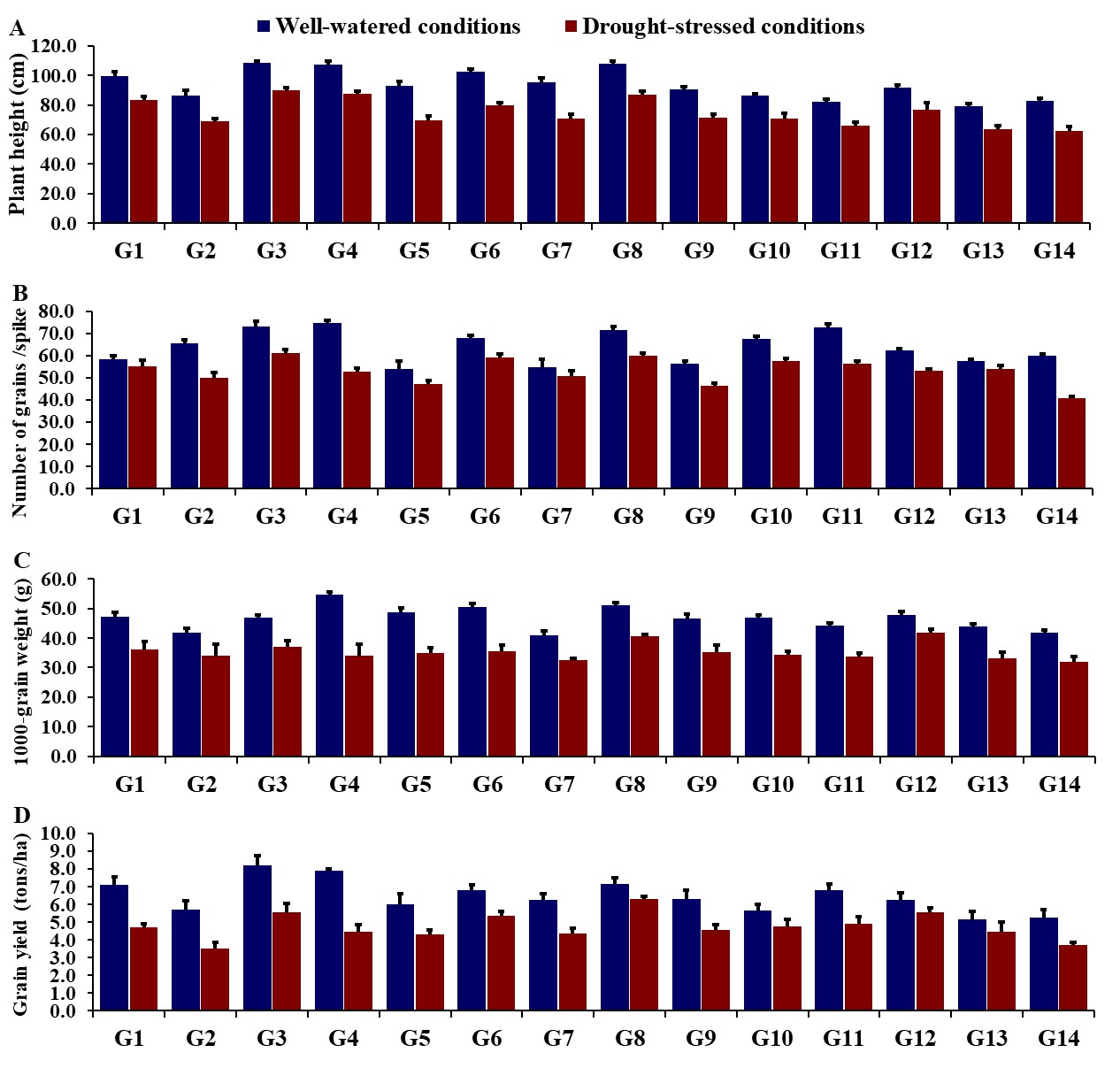
**Figure S4.** Comparative performance of evaluated wheat genotypes: (A): Plant height, (B): Number of grains/spike, (C): 1000-grain weight, and (D): Grain yield. The standard error (SE) is shown by the bars above the columns.
